# Beta-trace Protein as a new non-invasive immunological Marker for Quinolinic Acid-induced impaired Blood-Brain Barrier Integrity

**DOI:** 10.1038/srep43642

**Published:** 2017-03-09

**Authors:** Andreas Baranyi, Omid Amouzadeh-Ghadikolai, Dirk von Lewinski, Robert J. Breitenecker, Tatjana Stojakovic, Winfried März, Christoph Robier, Hans-Bernd Rothenhäusler, Harald Mangge, Andreas Meinitzer

**Affiliations:** 1 Department of Psychiatry and Psychotherapeutic Medicine, Medical University of Graz, Graz, Austria; 2 Institute for International Management Practice, ARU Cambridge, Cambridge, UK; 3 Hospital of the Brothers of St. John of God, Graz, Austria; 4 Division of Cardiology, Department of Internal Medicine, Medical University of Graz, Graz, Austria; 5 Department of Innovation Management and Entrepreneurship, Alpen-Adria-Universität Klagenfurt, Klagenfurt, Austria; 6 Clinical Institute of Medical and Chemical Laboratory Diagnostics, Medical University of Graz, Graz, Austria; 7 Synlab Academy, Synlab Services LLC, Mannheim, Germany; 8 Medical Clinic V (Nephrology, Hypertensiology, Endocrinology), Medical Faculty Mannheim, Ruperto Carola University Heidelberg, Mannheim, Germany

## Abstract

Quinolinic acid, a macrophage/microglia-derived excitotoxin fulfills a plethora of functions such as neurotoxin, gliotoxin, and proinflammatory mediator, and it alters the integrity and cohesion of the blood-brain barrier in several pathophysiological states. Beta-trace protein (BTP), a monomeric glycoprotein, is known to indicate cerebrospinal fluid leakage. Thus, the prior aim of this study was to investigate whether BTP might non-invasively indicate quinolinic acid-induced impaired blood-brain barrier integrity. The research hypotheses were tested in three subsamples with different states of immune activation (patients with HCV-infection and interferon-α, patients with major depression, and healthy controls). BTP has also been described as a sensitive marker in detecting impaired renal function. Thus, the renal function has been considered. Our study results revealed highest quinolinic acid and highest BTP- levels in the subsample of patients with HCV in comparison with the other subsamples with lower or no immune activation (quinolinic acid: F = 21.027, p < 0.001 [ANOVA]; BTP: F = 6.792, p < 0.01 [ANOVA]). In addition, a two-step hierarchical linear regression model showed that significant predictors of BTP levels are quinolinic acid, glomerular filtration rate and age. The neurotoxin quinolinic acid may impair blood-brain barrier integrity. BTP might be a new non-invasive biomarker to indicate quinolinic acid-induced impaired blood-brain barrier integrity.

## Quinolinic Acid

The inflammatory and neurodegeneration hypothesis of depressive illness considers major depression to be a psychopathological manifestation of inflammatory processes in the brain^1^,^2^. In this concept TNF-α, IFN-α and IFN-γ have a substantial impact on the enzyme indoleamine 2,3-dioxygenase (IDO), which causes a breakdown of the serotonin precursor tryptophan to kynurenine^1^,^2^. IDO is expressed in various cell types, including microglia, dendritic cells, monocytes and fibroblasts^2^. As a result, increased levels of kynurenine cross the blood-brain barrier and are subsequently broken up by human microglia into quinolinic acid, an excitotoxin with N-methyl-D-aspartate (NMDA) receptor affinity, and into other neurotoxic metabolites, which may trigger depressive symptoms^2^. Corresponding to this, Steiner *et al*.^3^,^4^ showed an upregulated production of quinolinic acid by microglia in the subgenual anterior cingulate cortex and the anterior midcingulate cortex in postmortem brains of patients with major depression who had committed suicide^3^,^4^.

The neuroactive metabolite of L-tryptophan quinolinic acid causes acute or chronic neuronal dysfunction through the following mechanisms: (a) Quinolinic acid is a potent NMDA receptor agonist and overstimulates the NMDA receptors in pathophysiological concentrations^5^,^6^,^7^. A massive entry of calcium into neurons, especially in the hippocampus, striatum and the neocortex might be the resulting consequence^5^,^6^,^7^,^8^. (b) Quinolinic acid causes excessive neurotoxic glutamate release by neurons and inhibits its reuptake by astrocytes^9^,^10^. (c) Quinolinic acid-induced reactive oxygen types mediate lipid peroxidation^11^,^12^,^13^,^14^,^15^,^16^.

(d) Quinolinic acid potentiates the toxicity of excitotoxins (e.g. glutamate, glycin, NMDA) and causes progressive mitochondrial dysfunction^17^. (e) Quinolinic acid impairs autophagy^18^. (f) Quinolinic acid destabilizes the cytoskeleton and intermediates filament hyperphosphorylation^19^,^20^,^21^. (g) Quinolinic acid plays an important role in the dysregulation of astroglial function and gliotoxicity^22^,^23^. Furthermore, Guillemin *et al*.^18^ showed that quinolinic acid selectively induces apoptosis of human astrocytes, which produce neuroprotective kynurenic acid. This might lead to lower neuroprotective action against neurotoxic quinolinic acid^24^,^25^. (h) Free radical production and oxidative stress are the consequence of quinolinic acid-induced NOS activity in astrocytes^18^,^24^. (i) Quinolinic acid causes a disruption of the integrity of the blood-brain barrier^4^,^18^,^26^,^27^.

In a study by Schefold *et al*.^28^ serum levels of kynurenine, kynurenic acid and quinolinic acid increased with chronic kidney disease severity (stages 4, 5 versus controls).

## Blood-Brain Barrier

Relating to anatomy, the brain’s microvascular network consists of capillaries, arterioles and venules and forms a protective blood-brain barrier (BBB) that separates the central nervous system (CNS) from the rest of the body, provides a homeostatic environment for the CNS, and restricts the exchange of material between the blood and the perivascular, extracellular fluid^29^,^30^,^31^. The capillaries form the largest and tightest microvasculature in the brain, while venules have a looser junctional arrangement^32^. The BBB strictly regulates the transport of blood-borne substances into the brain and is constituted of endothelial cells interconnected by a continuous line of tight junctions and of pericytes that are located in the duplication of the basement membrane^29^,^30^,^31^,^32^. In addition, the capillaries in the brain are surrounded by specialized structures of astrocytes^32^. Together these anatomical structures form the neurovascular unit (NVU) and incorporate a paracellular barrier (tight junctions, apical junctional complex), a transcellular barrier (low grade of trans- and pinocytosis), and an enzymatic barrier (metabolizes biologically active substances)^32^. In this way, the BBB restricts neurotoxic mixtures and large polar substances from passively diffusing into the brain^29^,^30^,^31^. In addition, members of the ATP-binding cassette (ABC) transporter family remove actively lipophilic compounds and metabolic toxins^4^. Numerous studies revealed that supporting cells of the NVU release a broad range of soluble factors that induce and control barrier properties^29^,^31^. Imola *et al*.^32^ revealed morphological differences in the vasculature of different CNS regions.

## Beta-Trace Protein

The Beta-trace protein (BTP), first described in 1961 by J. Clausen, is a monomeric glycoprotein that belongs to the lipocalin superfamily^33^,^34^,^35^. BTP consists of 168 amino acids and has a low molecular weight of 23,000 to 29,000 Da, depending on the degree of glycosylation^34^,^35^,^36^. In 1993, an amino acid sequence determination revealed that BTP has a prostaglandin synthase activity (prostaglandin-H2 D-isomerase; EC 5.3.99.2) and catalyses the conversion of prostaglandin H2 (PGH2), a common precursor of various prostanoids, to prostaglandin D_2_ (PGD2)^33^,^34^,^35^,^36^. PGD2 is involved in sleep induction and regulation, adipocyte differentiation, nociception, bronchoconstriction, inflammatory mediator modulation, nitric oxide release, induction of vasodilation, and inhibition of platelet aggregation^33^,^35^,^36^,^37^,^38^. In addition, Hoffmann *et al*.^39^ described localized and specific functions of BTP for the establishment and the function of the blood-tissue barriers.

BTP is brain-specific, one of the major polypeptide constituents of the cerebrospinal fluid (CSF), and is found in much lower concentrations in the blood^38^,^39^. The main origin of serum BTP has been hypothesized to result from diffusion of CNS BTP. After diffusion the liver rapidly eliminates the non-sialylated “brain type” glycoforms by specialized receptors, resulting in larger “blood/urine” sialylated glycoforms^38^. Corresponding with this, in canines intrathecally administered recombinant BTP was recovered from their serum and urine^38^. In the brain BTP is secreted to the major site by the leptomeninges and to a certain degree to the choroid plexus^34^,^35^,^40^,^41^,^42^. Previous studies showed that the low ventricular CSF concentration (1.5 mg/l) of beta-trace, which derives from the choroid plexus, increases along the CSF flow way in the subarachnoid space up to a mean lumbar concentration of 16.6 mg/l due to of a steady release from the leptomeninges^39^,^43^. In another study by Reiber *et al*.^41^ the mean BTP concentrations of 132 control patients were 18.4 mg/l in the normal lumbar CSF and 0.59 mg/l in the normal serum. CSF fistulas might be a severe consequence of head trauma, high intracranial pressure, arachnoid granulations, malignancy, surgical procedures at the skull base, or congenital malformation^34^,^35^. The detection of BTP to identify a suspected posttraumatic, iatrogenic, spontaneous or idiopathic cerebrospinal fluid leakage (CSF) with CSF rhinorrhea or otorrhea is a non-invasive, highly sensitive and specific method^34^,^35^,^40^,^41^,^42^,^43^,^44^,^45^. Furthermore, patients with an acute traumatic CSF leakage without dural repair have a high risk of contracting bacterial meningitis, a potential live-threatening disease^35^,^45^,^46^,^47^.

Additionally, changes in BTP urine and serum concentrations have been observed in renal diseases^48^,^49^. Thus, the kidneys eliminate BTP by glomerular filtration, and renal failure patients have increased serum BTP values^35^,^50^,^51^,^52^,^53^. Most recently, BTP has emerged as a novel biomarker of cardiovascular risk, and different studies have explored the role of BTP in hypertension^54^,^55^,^56^,^57^. Thus, increased BTP levels might also reflect injuries in the renal tubules and arterioles due to extended hypertension^58^,^59^.

## Aims of the Study

Quinolinic acid fulfills a plethora of physiological functions such as neurotoxin, gliotoxin, prooxidant molecule and proinflammatory mediator, and it alters the integrity and cohesion of the blood-brain barrier^26^,^27^. BTP is brain-specific and one of the major polypeptide constituents of the cerebrospinal fluid, and its detection to identify a suspected cerebrospinal fluid leakage is a well-established diagnostic method^35^. As a consequence, the prior aim of this study was to evaluate for the first time whether BTP might be a new non-invasive immunological marker for quinolinic acid-induced impaired blood-brain barrier integrity. We tested our research hypothesis in three subsamples with different conditions of immune activation and subsequent expected different quinolinic acid levels. BTP has also been described as a sensitive marker in detecting impaired renal function and serum levels of quinolinic acid increase with chronic kidney disease severity^28^. Thus, the renal function, including the glomerular filtration rate (GFR), were considered.

## Methods

### Participants and Study Design

To verify the hypothesis that BTP might be an innovative new non-invasive marker for quinolinic acid-induced impaired blood-brain barrier integrity, we examined the relations of quinolinic acid and BTP in three subsamples of our research project on quinolinic acid and kynurenine pathway^1^,^60^,^61^. The patient subsamples and the subsample of healthy controls were used to test the relations of BTP and quinolinic acid in different conditions of quinolinic acid levels. Thus quinolinic acid levels may differ in the two observed patient samples (patients with chronic hepatitis C virus (HCV) infection and IFN-α therapy; patients with major depression) and the sample of healthy controls due to the different conditions of immune activation. In detail, the first patient sample consisted of 41 patients with chronic HCV infection who were examined one month after the start of IFN-α therapy. All these HCV patients were treated on an ambulatory basis at the Division of Gastroenterology and Hepatology, Medical University of Graz, Austria. This subgroup was chosen because it is already known that IFN-α has a substantial impact on indoleamine 2,3-dioxygenase (IDO) that causes a breakdown of tryptophan to kynurenine and subsequently strongly increases quinolinic acid levels^1^,^60^,^61^. Thus we expected the highest quinolinic acid levels in this sample. The second sample consisted of 61 patients with major depression at the time of hospital admittance to the Department of Psychiatry, Hospital of the Brothers of St. John of God, Graz, Austria. In comparison to the sample with HCV infection and INF-α therapy, the immune activation associated with major depression and the subsequent quinolinic acid levels were expected to be much lower in the sample of patients suffering from major depression. Finally, the third sample included 45 physically and mentally healthy controls without a former history of psychiatric disorders. In this sample of healthy controls, lower quinolinic acid concentrations in comparison with patients during IFN-alpha therapy might be expected, due to the lack of concomitant immune activation. The details of the recruitment procedure are described elsewhere^1^,^60^,^61^. Exclusion criteria for all participants were (1) pregnancy, (2) significant comorbid conditions, (3) diagnosis of a neurological disease, and (4) for the patients with HCV infection, a coincidence with other chronic liver diseases.

Our research project and the current study have been approved by the Institutional Review Board of the Medical University of Graz.

Data protection met the standards set by Austrian law. The methods were carried out in accordance with the approved guidelines. All participants in this study had to give signed informed consent, and subjects could decide to withdraw from this study at any time.

### Biological Assessments

For all fasting study participants blood was sampled between 08.00 am and 09.00 am for the determination of quinolinic acid, creatinine and BTP.

Quinolinic acid was measured with a new developed liquid chromatography tandem mass spectrometry method^62^. After solid phase extraction and precolumn derivatization with butanol, analytes were separated on a reversed phase column and detected in the positive electrospray ionization (ESI) mode with the specific transition for quinolinic acid m/z 280 → m/z 78. Commercially available quinolinic acid -d3 was used as internal standards. Within-day CVs for quinolinic acid were 4.5% (225 nmol/L) and 1.2% (725 nmol/L), and between-day CVs were 7.2% (235 nmol/L) and 6.3% (752 nmol/L)^62^.

Concentrations of beta-trace protein were measured in serum with a latex-enhanced immunonephelometry method on a BN™ System (Siemens Healthcare, Erlangen, Germany). Creatinine measurement was done with an enzymatic method applied to an automated analyser (Cobas Mira Roche, Roche Diagnostics, Basel, Switzerland).

### Psychiatric Assessments, Sociodemographic Questionnaire, and Clinical Characteristics

The consenting participants of all subsamples were interviewed by experienced consultant-liaison psychiatrists (A.B., O.A.) to examine the presence and the severity of depressive symptomatology by using the well-validated psychometric observer-rated scale Hamilton Depression Rating Scale (HAMD-17). Recorded sociodemographic variables comprised age, sex, marital and employment status, and living arrangements. Clinical and treatment characteristics of the patients with HCV infection were: subtype of chronic HCV infection, type of IFN-α, and liver fibrosis as captured by fibroscans in kPA.

### Statistical Analyses

All descriptive statistics regarding sociodemographic and biochemical data are presented as mean and standard deviation (S.D.). χ^2^ tests were used to evaluate group differences in sociodemographic categorical variables. To test the data for normality we performed the Kolmogorov-Smirnov test. Log transformations for quinolinic acid and BTP were applied to make these data conform more closely to the normal distribution. Subsequently, for continuous measures, differences between groups were assessed based on the statistical distribution of variables using either one-way analyses of variance (ANOVAs) followed by Scheffé post hoc tests or Kruskal-Wallis H-Test followed by post hoc test with Bonferroni adjustment. By means of multivariate analyses in the form of a two-step hierarchical linear regression model, quinolinic acid was selected to predict beta-trace protein levels. Age, sex, group and renal function were chosen as control variables. In our analysis we considered sex and group as categorical variables. Therefore, we measured sex with one dummy variable taking the value 1 if the patient was male and the value 0 if the patient was female. For patient groups we formed two dummy variables. One dummy variable covered the subsample of patients with chronic HCV infection (subsample 1) and the second dummy variable was measured if the participant was from the subsample of patients with major depression (subsample 2). Thus, the healthy controls (subsample 3) formed the reference category in our analysis. In the subsample of of HCV patients Spearman’s rank correlations were performed to evaluate a potential association between the fibroscans in kPA and quinolinic acid as well BTP. All statistical analyses were performed with IBM SPSS Statistics 22.0 for Windows. P-values < 0.05 were regarded as statistically significant.

## Results

### Sociodemographic, Clinical and Treatment Characteristics

Table 1 provides the sociodemographic and clinical characteristics (including Hamilton Depression Rating Scale [HAMD-17] scores) of all study participants. All consenting 147 participants (60.5% women, 39.5% men) were Caucasian. In the sample of patients with major depression 58 (95.1%) patients had a medication-based therapy with selective serotonin re-uptake inhibitors or second-generation dual-action antidepressants. Two patients (3.3%) had a medication-based therapy with a tricyclic antidepressant and one patient (1.6%) had an additional pharmacological treatment with carbamazepine. The sample of patients with HCV had a medication-based therapy with Peg INF-α-2 orPeg INF-α-2b. In the sample of healthy controls no patient had a psychopharmacological treatment or a Peg INF-α therapy.

### Beta-Trace Protein and Quinolinic Acid

In a two-step hierarchical linear regression model using the ordinary least squares (OLS) estimator with heteroscedasticity-consistent (robust) standard errors quinolinic acid was selected to predict beta-trace protein levels. Age, sex (female = 0; male = 1), group [HCV- patients one month after the start of IFN-α therapy (dummy variable 1); patients with major depression (dummy variable 2); healthy controls (reference group)] and renal function (GFR) were chosen as control variables.

In the first step, we regressed beta-trace protein levels against the control variables age, sex, group and renal function (Model 1). This model yields a R^2^adj. = 0.297 and is statistically highly significant (p < 0.001). In the second step we introduced the predictor quinolinic acid (Model 2). This model is also highly significant and yields a R^2^adj. = 0.372; p < 0.001. Additionally, the ∆ R^2^ = 0.077 is highly significant (p < 0.001), which means that the introduction of quinolinic acid to the model significantly improves the explanation of the variance of BTP. In this step, the model shows that significant predictors of beta-trace protein levels are quinolinic acid levels, GFR and age. Table 2 provides the results of the two-step hierarchical linear regression model. (In addition, we tested for group effects concerning the effect of quinolinic acid levels by including interaction effects of quinolinic acid with group dummy variables. Both the overall improvement of the model (∆ R^2^ = 0.006, p = 0.475) as well as the interaction effects (quinolinic acid*Group-HCV: p = 691; quinolinic acid*Group-Major Depression: p = 0.378) were not significant and therefore lead to no model improvement).

One-way analyses of variance (ANOVAs) additionally showed that patients with HCV infection and IFN-α therapy had significantly higher quinolinic acid levels in comparison to patients with major depression as well as healthy controls. Corresponding with this, the BTP levels differed in the same way. Thus, patients with HCV infection and IFN-α therapy had the highest BTP levels. Table 3 shows the biological assessments.

In the subsample of HCV patients, liver fibrosis as captured by fibroscans in kPA did neither correlatewith quinolinic acid (Spearman’s rank correlation coefficient r: −0.091, p = 0.729) nor beta-race protein levels (Spearman’s rank correlation coefficient r: −0.018, p = 0.944). Table 1 provides the fibroscan data (Supplementary Information).

## Discussion

Several former studies have established an impact of quinolinic acid on blood-brain barrier integrity. According to Heyes *et al*.^63^, under normal conditions quinolinic acid in the blood contributes to almost 70% of this metabolite in the CSF, while in conditions of immune activation, the elevations of CSF quinolinic acid indicate both de novo synthesis within the brain as well as entry of quinolinic acid from the blood due to defects in the integrity of the BBB. In a study of Reynolds and Morton^27^ trypan blue was used to show increases in BBB permeability in striatal lesions induced by intrastriatal injection of quinolinic acid. Diverse research studies in the past have investigated that increased CSF of quinolinic acid may also reflect abnormal blood–brain barrier function in patients with acquired immunodeficiency syndrome (AIDS) dementia complex, exhibiting a relationship to their clinical and neurological status^26^. In a study by St’‘astný *et al*.^26^ potential BBB dysfunction was explored in young adult male Wistar rats four days after the intracerebroventricular infusion of quinolinic acid by measuring plasma albumin extravasation using rocket immunoelectrophoresis. In this study, the intracerebroventricular infusion of quinolinic acid failed to raise the extracellular tissue concentration of albumin in the entorhinal cortex. However, significantly higher levels were detected in the hippocampus proper (but not in the subiculum region and dentate gyrus) and in the striatum^26^. In addition, Ryu *el al.*^64^ showed that an extensive disruption of BBB was observed at 7 days’ post quinolinic acid-injection as demonstrated by increased immunohistochemical staining using an antibody against immunoglobulin G (IgG). The study results also revealed that quinolinic acid intrastriatal injection resulted in significant increases in the number of infiltrating T-lymphocytes (by 70-fold) and expression of major histocompatibility complex (MHC-class II) (by 45-fold) relative to unlesioned controls.

Our study results revealed the highest BTP and quinolinic acid levels in the subsample of patients with HCV infection and INF-α therapy in comparison with the other subsamples with low or no immune activation. In this subsample the observed elevated quinolinic acid levels might be the result of increased immune activation due to INF-α, resulting in increased levels of kynurenine, which crosses the blood-brain barrier and is subsequently broken up by human microglia into quinolinic acid. The simultaneously elevated BTP might be a strong indicator that in somatic conditions with high quinolinic acid levels BTP might be a new non-invasive marker of quinolinic acid-induced impaired blood-brain barrier integrity. Corresponding with this, the two-step hierarchical linear regression model revealed that quinolinic acid is a highly significant predictor of BTP in all examined subsamples. Furthermore, impaired GFR and older age have the already expected additional impact on BTP levels, while liver fibrosis had no significant impact on quinolinic acid or BTP. In conclusion our study results reveal that participants with high levels of neurotoxic quinolinic acid also have higher BTP levels that might indicate CSF leakage.

### Limitations

Peripheral markers of BBB function have been known for many years and their clinical use has confirmed their utility. Usually, clinicians refer to the beta-trace protein (BTP) serum concentration to evaluate severe traumatic injuries of the BBB with liquorrhea. In these cases, BTP serum concentrations are elevated in comparison with the respective BTP concentration in aural or nasal secretion. However, so far no meaning has been attached to the physiological fluctuation of BTP. However, it is quite conceivable that even marginally increased BTP values indicate a loss of selectivity of the BBB, which could have an impact on neurophysiological processes and, as a consequence, on physical and mental health. Our study results are a first hint that BTP might also be a non-invasive diagnostic peripheral marker of BBB permeability. A direct comparison with S100B or similar markers should be performed in subsequent studies. Furthermore, it should also be considered to investigate in neuroimaging studies whether marginally increased BTP serum values can be correlated with signs indicating an impairment of BBB permeability. It also seems reasonable to integrate it in cohort studies to detect its possible clinical relevance for neurological and neuropsychological diseases. Thus, additional research studies are required to determine any set patterns of this present exploratory study. Knowledge about the complex interaction between BTP and quinolinic acid is still limited and requires ongoing research.

## Conclusions

Several studies demonstrated that the neurotoxin quinolinic acid might impair blood-brain barrier integrity. As a consequence, new non-invasive biomarkers might be of great scientific and clinical value to assess the physiological consequences of increased quinolinic acid levels in the human brain. BTP might be such a new appropriate non-invasive biomarker to indicate quinolinic acid-induced impaired blood-brain barrier integrity.

## Additional Information

**How to cite this article:** Baranyi, A. *et al*. Beta-trace Protein as a new non-invasive immunological Marker for Quinolinic Acid-induced impaired Blood-Brain Barrier Integrity. *Sci. Rep.* **7**, 43642; doi: 10.1038/srep43642 (2017).

**Publisher's note:** Springer Nature remains neutral with regard to jurisdictional claims in published maps and institutional affiliations.

## Supplementary Material

Supplementary Dataset 1

## Figures and Tables

**Table 1:**
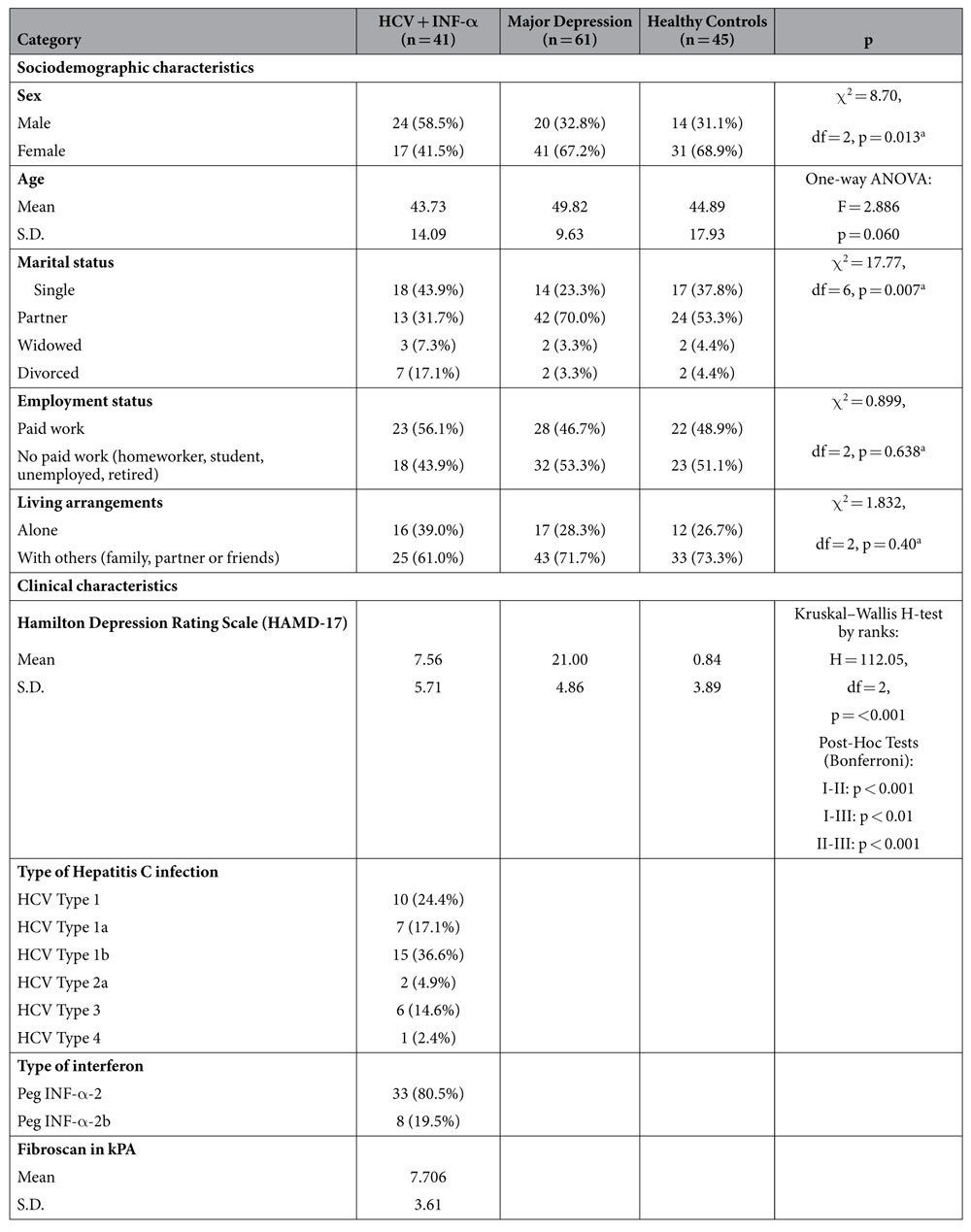
Sociodemographic and clinical characteristics of the participants.

S.D. = Standard deviation.

^a^χ^2^ tests.

**Table 2:** Two-step hierarchical linear regression model.

DV = Beta-Trace Protein (BTP) Total Sample (n = 147)					
Step 1 (Model 1)	B	β	S.E.	p	VIF
Constant	−0.186		0.061	0.003	
Sex –male (Dummy variable)	0.020	0.105	0.014	0.148	1.083
Age	0.001	0.224	0.001	0.008	1.432
Group^a^ - HCV (Dummy variable 1)	0.050	0.245	0.017	0.004	1.486
Group^a^ - Major Depression (Dummy variable 2)	−0.004	−0.020	0.015	0.808	1.415
Renal Function - Glomerular Filtration Rate	−0.002	−0.310	0.000	0.000	1.389
	F = 13.32***; R^**2**^** = 0.321; adjusted R**^**2**^** = 0.297**				
**Step 2 (Model 2)**					
Constant	−0.686		0.131	0.000	
Sex –male (Dummy variable)	0.015	0.082	0.013	0.236	1.090
Age	0.001	0.188	0.001	**0.019**	1.449
Group^a^ - HCV (Dummy variable 1)	0.027	0.133	0.017	0.116	1.647
Group^a^ - Major Depression (Dummy variable 2)	0.007	0.039	0.015	0.622	1.460
Renal Function - Glomerular Filtration Rate	−0.001	−0.251	0.000	**0.002**	1.434
Quinolinic Acid	0.185	0.331	0.044	**0.000**	1.423
	F = 15.418***; R^**2**^** = 0.398; adjusted R**^**2**^** = 0.372**				
	**∆ R**^**2**^ = 0.077***				

Standardized regression coefficients are displayed in the table; Significance Levels: *p < 0.05; **p < 0.01; ***p < 0.001; Log 10 transformations for quinolinic acid and BTP; ^a^Group: HCV patients one month after the start of IFN-α therapy (Dummy variable 1); patients with major depression (Dummy variable 2); healthy controls (reference group).

**Table 3:** Biological Assessments.

	I: HCV patients + INF-α (n = 41)	II: Major Depression (n = 61)	III: Healthy Controls (n = 45)	p
Beta-Trace Protein (BTP) [mg/L]	Mean: 0.619 S.D. = 0.122	Mean: 0.543 S.D. = 0.109	Mean: 0.539 S.D. = 0.119	**One-way ANOVA:** F = 6.792 p = 0.002 **Post-Hoc Tests (Scheffé):** I-II: p = 0.007 I-III: p = 0.005 II-III: p = 0.951
Quinolinic Acid [nmol/L]	Mean: 563.11 S.D. = 161.121	Mean: 371.23 S.D. = 125.84	Mean: 428.30 S.D. = 202.08	**One-way ANOVA:** F = 21.027 p < 0.001 **Post-Hoc Tests (Scheffé):** I-II: p < 0.001 I-III: p < 0.001 II-III: p = 0.208
Creatinine [mg/dL]	Mean: 0.872 S.D. = 0.147	Mean: 0.814 S.D. = 0.180	Mean: 0.855 S.D. = 0.181	**One-way ANOVA:** F = 1.585 p = 0.209
Glomerular Filtration Rate[mL *min^−1^]	Mean: 87.44 S.D. = 13.83	Mean: 91.70 S.D. = 15.41	Mean: 92.63 S.D. = 18.45	**One-way ANOVA:** F = 1.295 p = 0.277

S.D. = Standard deviation; Log 10 transformations for quinolinic acid and BTP.
